# Integrated radiometric and mineralogical investigation of radioactive mineralization in the El-Erediya Shear Zone, Eastern Desert, Egypt

**DOI:** 10.1038/s41598-025-27562-0

**Published:** 2025-12-01

**Authors:** Hassan Rageh, Mahmoud Abdel-Hakeem, Mohamed El-Tahir, Ehab Abu Zeid, Maurizio Milano, Mahmoud Ahmed Abbas

**Affiliations:** 1 Geology Department, Faculty of Science, South Valley University, Qena, 83523 Egypt; 2 Nuclear Materials Authority, Maadi, Cairo, 11511 Egypt; 3 Department of Earth, Environment and Resources Sciences (DiSTAR), University of Naples Federico II, 80126 Naples, Italy

**Keywords:** El-Erediya shear zone, Radioactive minerals, Radioelement anomalies, FCM, Ore microscopic, SEM–EDX analysis, XRD analysis, Environmental sciences, Natural hazards, Solid Earth sciences

## Abstract

The shear zone cut through the western southern part of El-Erediya granite pluton represents one of the most promising radioactive minerals occurrences in Egypt’s Eastern Desert. To explore this shear zone, exploratory tunnels were dug at wadi level. The present study aims to map the potential mineralization, determine geological units, and their relations with mineralizations along the main adit and shear zone of El-Erediya exploratory tunnels. Fuzzy C-means analysis (FCM) is applied to measured ground gamma ray spectrometry data (eU, eTh, and K^40^). FCM identified three distinct radiometric clusters, revealing the spatial association of radioelement enrichment. The variation in the occurrence of different geological features within these clusters indicates that post-magmatic processes significantly influence the distribution and concentration of radioelements throughout the study area. Mineralogical investigations identified the occurrences of pyrite, chalcopyrite, arsenopyrite, marcasite, magnetite, goethite, hematite, uranophane, uranopilite, kasolite, betafite, plumbobetafite, ishikawite, thorite, zircon, and xenotime that have either magmatic or non-magmatic (hydrothermal and supergene) origins. Additionally, mineralogical investigations show that most of the radioactive minerals are found in cluster No. 2. These minerals are associated with opaque minerals (e.g., pyrite, hematite, goethite, pyrolusite, and fluorite), which serve as capturing agents for uranyl-ions from hydrothermal solutions. The integration of radiometric and mineralogical analysis provides an effective tool for identifying potential zones of radioactive mineralization and enhances understanding of their lithological controls.

## Introduction

The gamma-ray spectrometry method is one of the commonly used geophysical methods, whether airborne or ground, and has been widely used for many years in various fields, that measured total radioactivity and the surface distribution of naturally occurring radioelements (K^40^, eTh, eU). This method was developed as a tool for uranium exploration, while its applications now include: mineral exploration^1–3^, geological mapping^4–7^, soil mapping^8–13^, monitoring environmental radiation^14–17^, and geothermal exploration^18^. Wilford et al. confirmed that patterns from airborne gamma-ray spectrometry provided crucial insights for studies of soil, regolith, and geomorphology, which are essential for land management and mineral exploration decisions^9^. Moreover, gamma-ray spectroscopy provides a quick and dependable method for analyzing uranium and thorium ores^19^. Darnley and Ford demonstrated that in many cases, gamma-ray spectrometry is likely more effective than any other airborne geophysical or remote sensing technique for providing information that can be directly interpreted in terms of surface geology^20^. Regional geophysics airborne radiometric and magnetic surveys discovered several radioactive anomalies in the basement rocks around Qena-Safaga Road at the Central Eastern Desert of Egypt^21^. These anomalies were substantiated by ground surveys and systematic fieldworks and found that most of promising radiometric anomalies are found within the younger granite, especially El-Erediya pluton^22^. Moreover, these ground works revealed that these anomalies concentrated and associated with the shear zone located in the southern western part of El-Erediya pluton. This was followed by excavating exploratory tunnels at wadi level through anomalies portion of El-Erediya pluton for exploring and studying subsurface geology of the shear zones^23,24^. The previous ground radiometric studies of the subsurface within the shear zone were carried out by El-Tahir using Gamma-Ray Scintillometer (Model GR-101) which provides readings in counts per second (C.P.S)^23^, while other studies were performed in selected, limited parts by Abu-Deif et al. using Gamma-Ray Spectrometer GS256^25^. Depending on fact of the concentration and distribution of radioelements (U, Th, and K^40^) is controlled by type of lithology and geologic processes (weathering, erosion, and transportation)^20,26^, the current investigation aims to study the distribution of the radioelements along the shear zone along the exploratory tunnels and the fresh granite in the main adit of the tunnels and determine locations of radioelements anomalies that will be integrated with geology to delineate the zonations of the radioactive minerals. This study provides a comprehensive mineralogical and radiometric analysis of the El-Erediya shear zone, a key area for radioactive mineralization in Egypt’s Eastern Desert. By applying ground gamma-ray spectrometry combined with fuzzy C-means clustering, the study offers an effective approach to accurately map and classify subsurface radiometric anomalies. The integration of mineralogical investigations further clarifies the nature and origin of radioactive minerals within the shear zone. To achieve these objectives, ground radiometric (gamma-ray) measurements will first be performed through El-Erediya exploratory tunnels. The data collected will then undergo cluster analysis to classify areas based on their radioelement content. Verification of the anomalous zones will be carried out through petrographic, ore microscopic, and mineralogical investigations, as detailed in the next sections.

## Geological setting

**Fig. 1:**
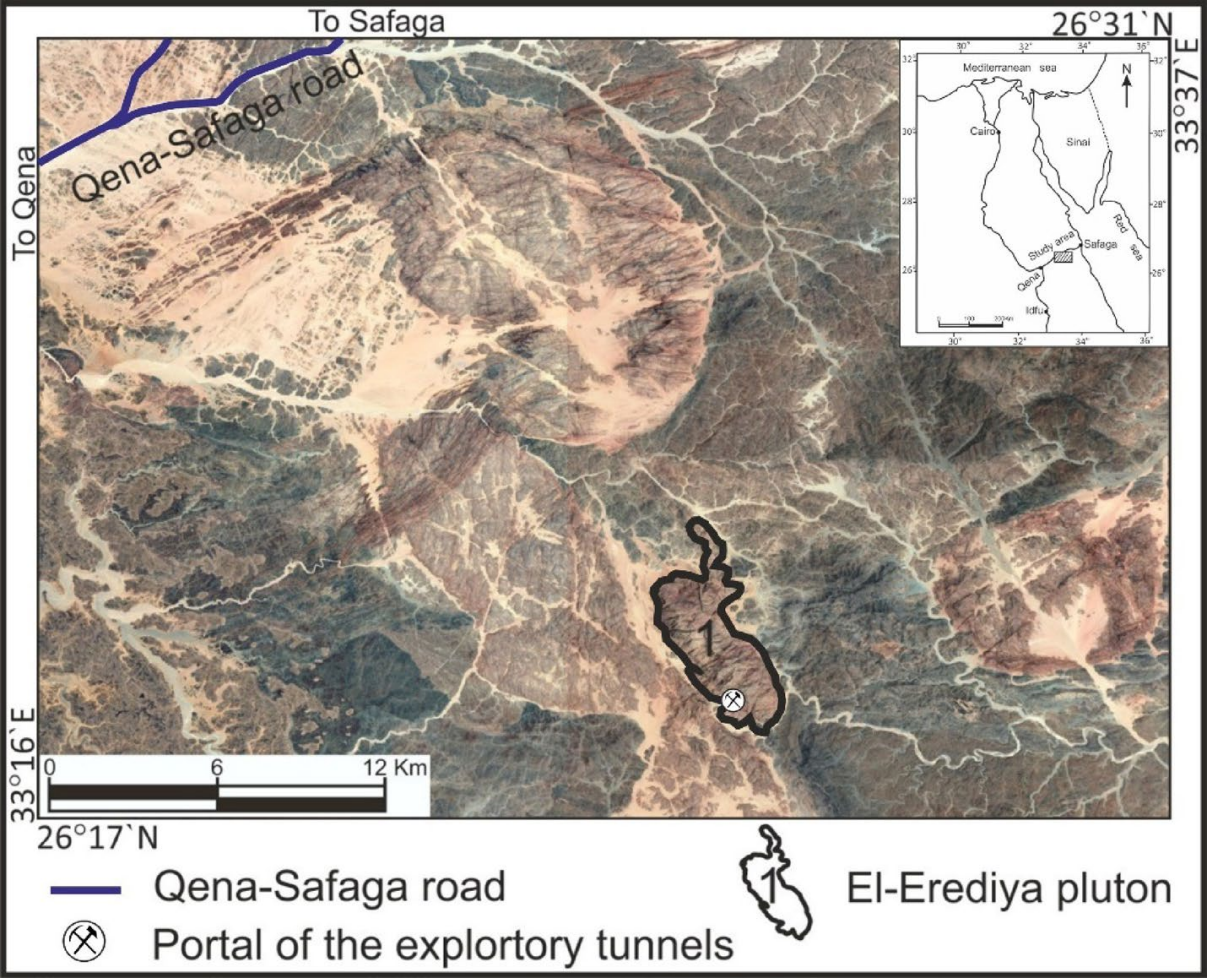
Location map (2025 Google) of El-Erediya pluton showing the portal of the exploratory tunnels.

**Fig. 2:**
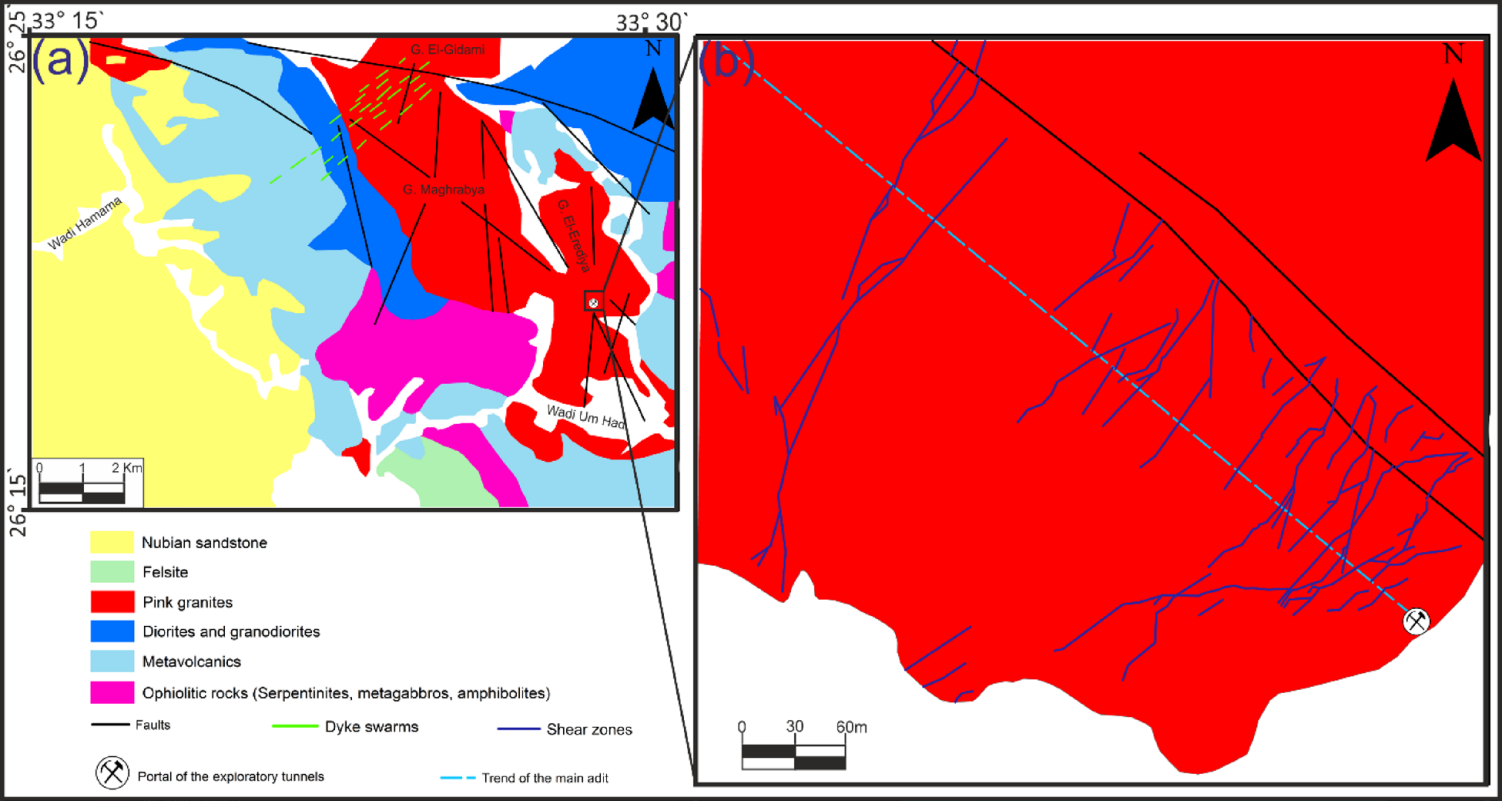
(**a**) Geologic map of El-Erediya area (modified from Abu-Deif 1993) and (**b**) Detailed geological map of the surface of the El-Erediya pluton (modified after El-Tahir, 1985).

**Fig. 3:**
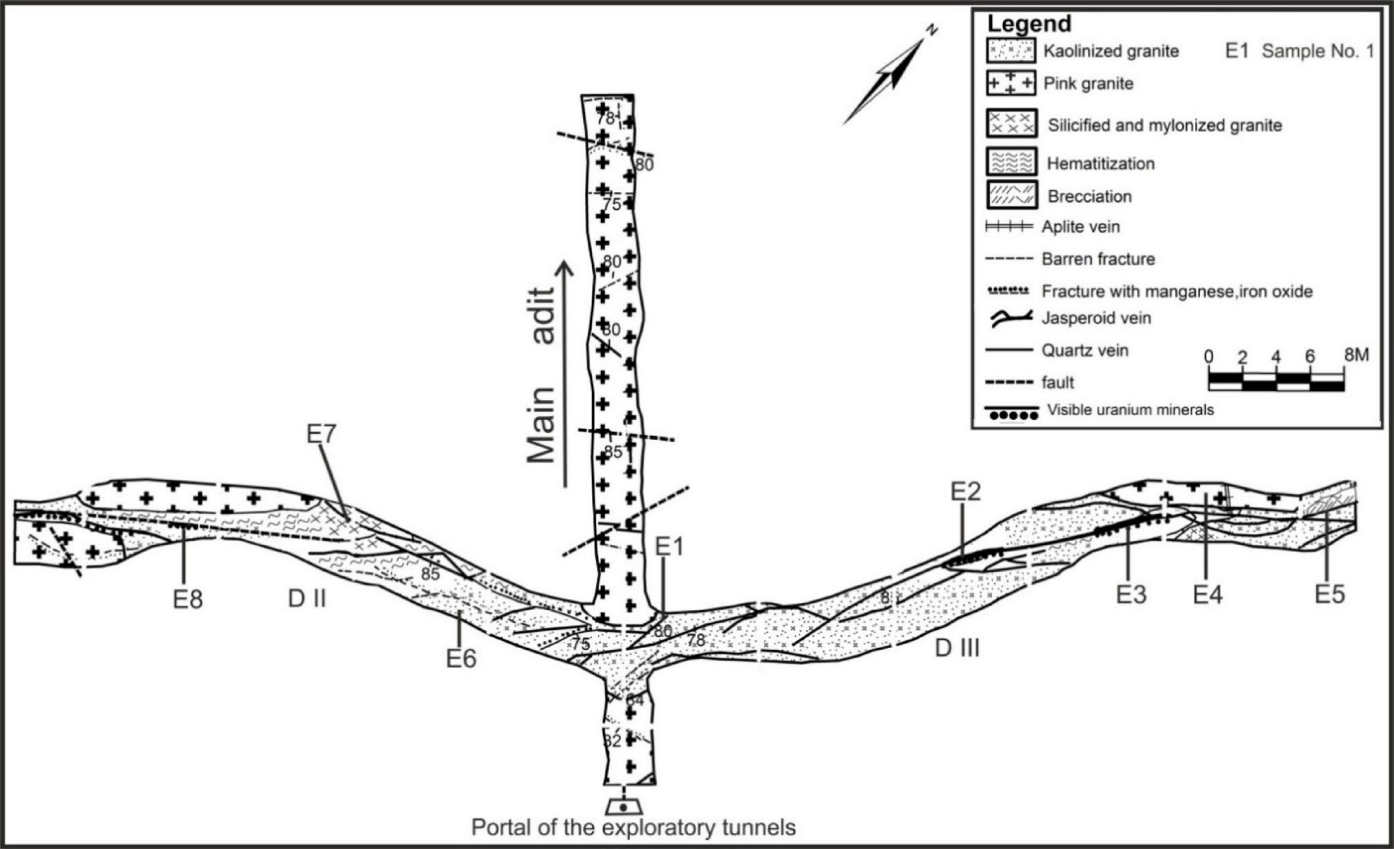
Geologic maps of drifts# DII&DIII through the shear zone#2 of El-Erediya exploratory tunnels (after El-Tahir, 1985), and locations of collected representative samples.

**Fig. 4:**
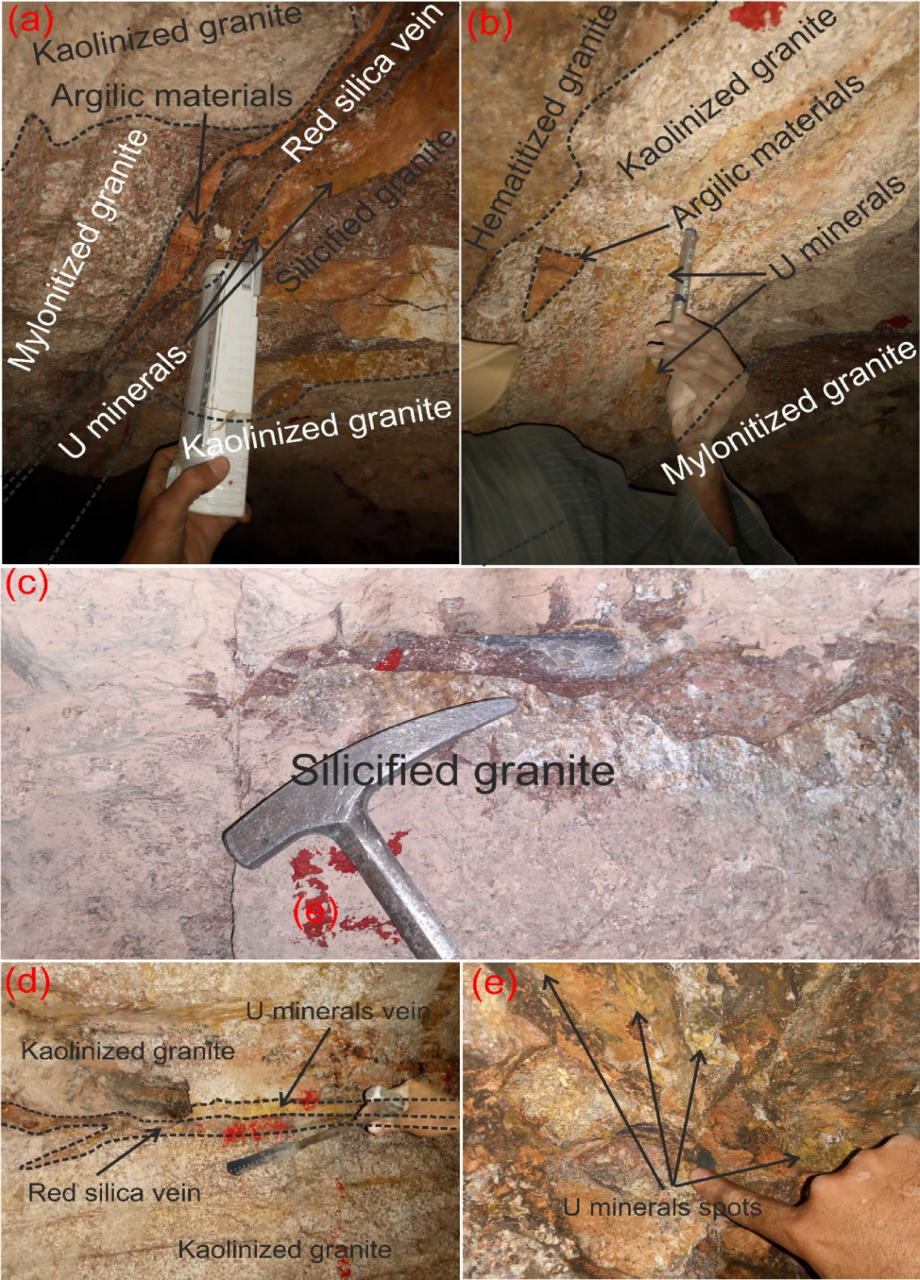
Field photographs show alteration of pink granite and visible uranium minerals through drift# DII and drift# DIII of El-Erediya exploratory tunnels (**a**) kaolinized granite, silicified granite, mylonitized granite, red silica vein, (**b**) hematitized granite, kaolinized granite, and mylonitized granite, silicified granite, (**d**) uranium mineral vein, and (**c**) uranium minerals spots.

El-Erediya pink granite is located in the Central Eastern Desert, between latitudes 26° 18′ 35″ and 26° 20′ 2″ N, and longitudes 33° 28′ 10″ and 33° 29′ 43″ E. It lies approximately 25 km south of the Qena-Safaga road near the 85 km mark (Fig. 1). It is exposed as an oval-shaped pluton that extends in NW direction parallel to the Red sea, with average length and width about 6.5 km and 2.5 km, respectively. This locality contains several rock units, arranged from oldest to youngest: ophiolitic rocks, island arc (metavolcanics), early magmatic phase (diorite and granodiorite), later magmatic phase of undeformed granites (pink granite), and felsite (Fig. 2a). El-Erediya pink granite was largely intruded into metamorphosed basic rocks of the ophiolitic-island arc sequence (Fig. 2). In some areas, the neighboring country rocks were undergone thermal metamorphism at their contact with the granite. This intrusion happened during the post-tectonic period in Egypt, around 600 Ma^27^. The Rb–Sr age for El-Erediya is 570 ± 5 Ma^28^, while the U–Pb age is reported as 583 ± 21 Ma^29^. The geology of the El-Erediya pluton and its hosted vein-type U mineralizations have been a topic of extensive discussion by several authors^22–25,29–51^. The El-Erediya pluton is characterized as a massive, greyish pink to pink colored granite that is medium-to-coarse-grained. It is mainly composed of perthite (31–34%), plagioclase (31–35%), and quartz (30–35%), with smaller amounts of biotite (1–2%), muscovite (0.5–1%), and opaque minerals (0.5–1%). Additionally, it contains accessary minerals such as zircon, apatite, monazite, fluorite, sphane and garnet. Geochemically, El-Erediya granite is slightly peraluminous^47^ and post-orogenic, with high content of silica and alkaline elements (K_2_O and Na_2_O) and low content of (MgO, Fe_2_O_3_ and TiO_2_)^47^. This granite is also rich in U, Rb, Nb and Y but depleted in Sr, Pb, Ba Zr and Zn^37^. Structurally, El-Erediya pink granite is intersected by NE trending shear zones, NW trending faults, dykes, veins of aplite, porphyry, pegmatite, and a few basaltic dikes. Moreover, it is bounded to the NE and SW sides by faults that trend NW. On the other hand, the exploratory tunnels in the southern part of El-Erediya plutons (Fig. 3) showed that the pink granite is cut by various fractures at the main adit and has undergone hydrothermal alteration including kaolinization, silicification, hematitization, mylonitization, sericitization, as well as some black manganese oxides and argillic materials along the extension of the shear zone (Fig. 4). The center of the shear zone is occupied by parallel sets of red silica veins, containing SiO_2_ (87.5 wt %), Fe_2_O_3_ (5.37 wt %), Al_2_O_3_ (4.77 wt %), K_2_O (1.57 wt %), F (1200 ppm), U (758 ppm), Ba (274 ppm), Pb (240 ppm), Zr (168 ppm), Zn (148 ppm), Th (20 ppm)^40^ that mainly directed in NE-SW, with minor directions of NW–SE, and N-S (see Fig. 2b). These veins host U mineralizations^22,33^, such as pitchblende, uranophane, autunite, and renardite, with thickness varying from a few centimeters up to 10 cm. The occurrence of Pitchblende is rare due to its highly unstable behavior under chemical weathering. Therefore, yellowish to brownish yellow secondary U minerals are commonly observed as thin veins and spots during fieldworks (see Fig. 4).

## Methodology

### Geophysical data (radiometric data) acquisition and processing

**Fig. 5:**
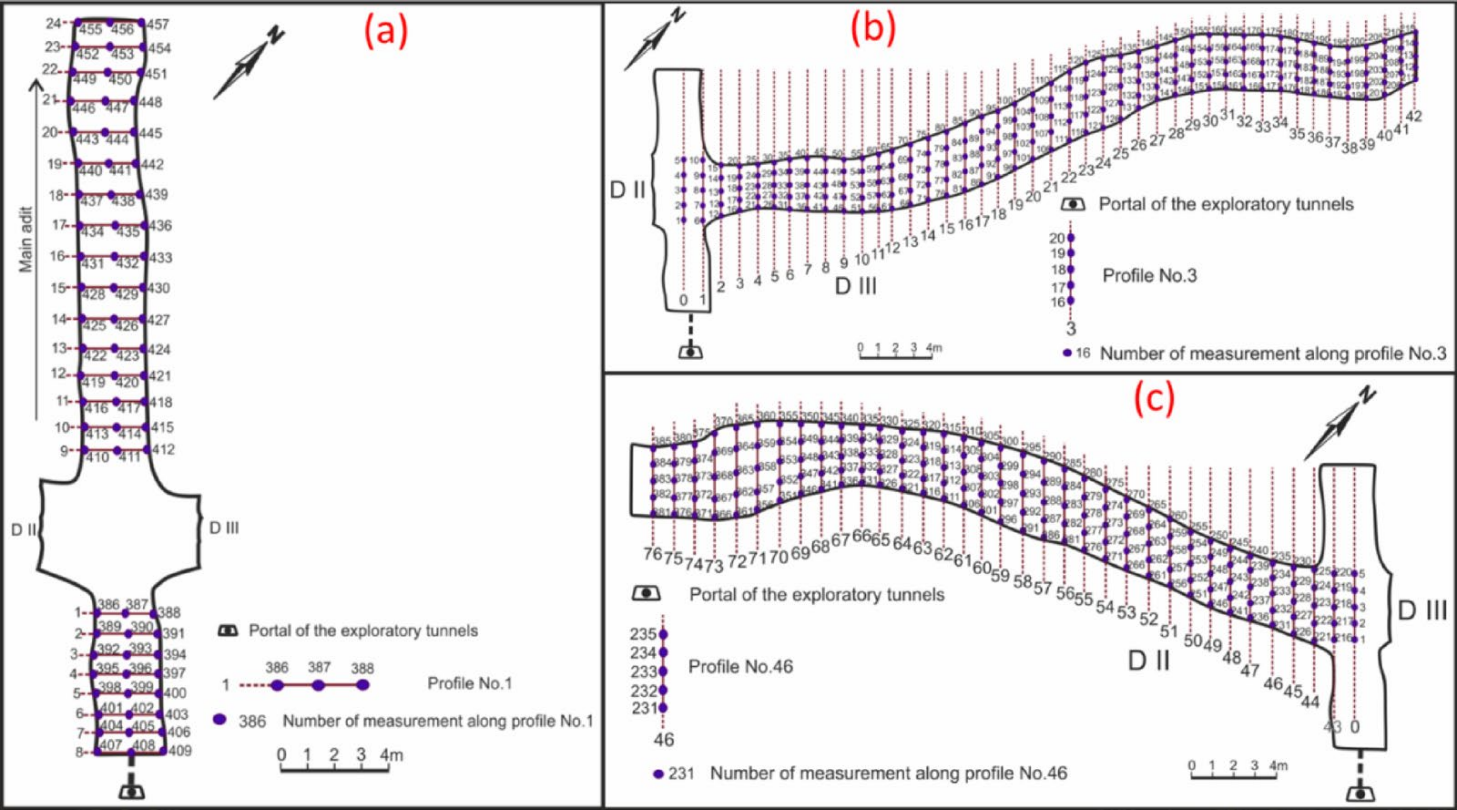
Locations of radioelement measurements at the roof of the main adit (**a**), drift# DIII (**b**), and drift# DII (**c**) of El-Erediya exploratory tunnels.

To explore and identify radioactive mineralization, ground radiometric measurements were conducted through El-Erediya exploratory tunnels by handheld Gamma-Ray Spectrometer, Model RS-230, with large BGO (Bismuth Germanate Oxide) detector. The instrument was calibrated by the manufacturer for measuring the three natural radioelement concentrations (eU (ppm), eTh (ppm), and K^40^ (%)). These measurements were recorded at the roofs of the main adit and the shear zones of El-Erediya (drifts No. II & III) by constructing a series of parallel profiles, spaced 1 m apart and oriented nearly perpendicular to the main direction of the adit and drifts. For each profile, three measurements were taken at the back (roof) of the main adit, while five measurements were taken along the back extension of the drifts (Fig. 5). The total number of these measurements was 72 for the main adit and 385 measurements for the shear zone, respectively. The data obtained from radioelement concentrations was processed statistically and mapped.

### Fuzzy C-means clustering analysis

Fuzzy C-Means clustering (FCM) is applied to the measured gamma rays data to integrate the different radioelement maps and provide a classification of lithological units in the El-Erediya exploratory tunnels. FCM is an unsupervised machine learning classification method used to identify patterns or structures in data without requiring prior expert input. The core aim of this technique is to increase the similarity of data points within the same cluster while reducing similarity between different clusters. The mathematical formulation of FCM is represented by the following objective function^52^:

1 $$J_{FCM} \left( {U, V} \right) = \mathop \sum \limits_{i = 1}^{c} \mathop \sum \limits_{k = 1}^{n} \left( {u_{ik} } \right)^{m} x_{k} - \upsilon_{i}^{2} ,$$

$${\text{dependent}}\;{\text{upon}}\; \mathop \sum \limits_{i = 1}^{c} u_{ik} = 1.$$

Here, c stands for the total number of clusters, *n* refers to the total number of the data points (*Χ*_1_, *Χ*_2_, *Χ*_3_, …, *Χ*_*k*_, …,*Χ*_*n*_), *m* designates weighting exponents (1 ≤ *m* < ∞), and *V* is the matrix containing the cluster center values (*υ*_1_, *υ*_2_,…, *υ*_*i*_,…, *υ*_*c*_). *U* denotes the association matrix wherein each element u_ⅈk_ stipulates the affiliation degree of the *k*th data point in the *i*th cluster. The notation ʽ||.||ʼ indicates the Euclidean norm, used to measure the similarity between a data point and a cluster center. The following formula can be used to represent the *i*th cluster’s center point (*υ*_*i*_).

2 $$\upsilon_{i} = \frac{{ \mathop \sum \nolimits_{k = 1 }^{n} \left( {u_{ik} } \right)^{m} x_{k} }}{{\mathop \sum \nolimits_{k = 1}^{n} \left( {u_{ik} } \right)^{m} }}$$

The ideal locations for cluster centers, which are anticipated to be found in regions with a significant number of sample data points, are determined by Eq. (2). Additionally, each member (*u*_*ik*_) of the *U* matrix can be found by the following equation.

3 $$u_{ik } = \left[ {\mathop \sum \limits_{a = 1}^{C} \left( {\frac{{d_{ik} }}{{d_{ak} }}} \right)^{{2/\left( {m - 1} \right)}} } \right]^{ - 1}$$

$${\text{Subject}}\;{\text{to}}\;d_{ik}^{2} = \left\| {x_{k} - \upsilon_{i} } \right\|^{2} .$$

Afterwards, taking into account the initial parameters, including the number of clusters (*c*), the weighting exponent (*m*), and an initial estimate for either the membership matrix (*U*) or the cluster center positions (*V*), the objective function of the Fuzzy C-Means clustering technique is minimized iteratively using an alternating optimization approach.

The raw gamma-ray spectrometry data undergo normalization to standardize the data by transforming it into a specified range using the Z-score standardization method, enhancing cluster quality and improving the accuracy of clustering algorithms. The Z-score standardization formula for a given set of raw data is defined as:

4 $$Z\left( {x_{ij} } \right) = \frac{{x_{ij} - \overline{x}_{j} }}{{\sigma_{j} }}$$

where $\overline{x}_{j}$ and $\sigma_{j}$ are sample mean and standard deviation, respectively. The transformed variable has a zero-mean and standard deviation of 1.

Consequently, the obtained clusters were validated by collecting eight representative samples from all clusters, followed by ore-microscopic investigation and radioactive minerals separation.

### Thin and polished sections

Petrographic and ore microscope investigations were carried out on representative samples from different clusters. Ten thin and polished sections were prepared at the laboratory of Cairo university. These were examined using a Polarized Transmitted and Reflected Light Microscope in the petrography lab of Nuclear Material Authority (NMA) to identify silicate minerals and their alterations, as well as opaque minerals.

### Mineral separation

Following petrographic and ore-microscopic studies, eight representative samples were subjected to mineral separation processes involving grinding the samples to a size of − 500 µm and conducting bromoform-bases separation, followed by handpicking under a Binocular Microscope to separate radioactive mineralization (heavy minerals).These heavy minerals were detected and analyzed by using an Environmental Scanning-Electron Microscope (ESEM model Philips XL30 and Prisma E), supported by an Energy Dispersive Spectrometer (EDX) unit, which was used at 25–30 kV accelerating voltage, 1–2 mm beam diameter, and 60–120 s counting time. The ESEM-EDX analyses were made for the individual mineral grains using a counting time of 180 s for some selected spots near the centers of the grains to avoid diffraction of the electron beam at the grain margins. The analytical conditions were 25–30 kv accelerating voltages, 1–2 mm beam diameter, and 60–120 s counting time. Minimum detectable weight concentration is from 0.1 to 1 wt. %. Precision is well below 1% while the relative accuracy of concentration measurements ranges from 2 to 10% for elements with Z > 9 (F) and from 10 to 20% for the lighter elements B, C, N, O and F. ESEM-EDAX analyses were also used to investigate morphological characteristics of these minerals as well as to give a semi-quantitative evaluation of their elemental composition. Oxygen and carbon are excluded from the analysis for their low precision. Additionally, the identified minerals were confirmed by X-Ray Diffraction (XRD) using powder diffractometer (Brucker D8 Advance, Germany in the range of 10° to 70°) in the Nuclear Material Authority’s lab.

## Results and discussion

### Gamma rays radioelements maps

**Fig. 6:**
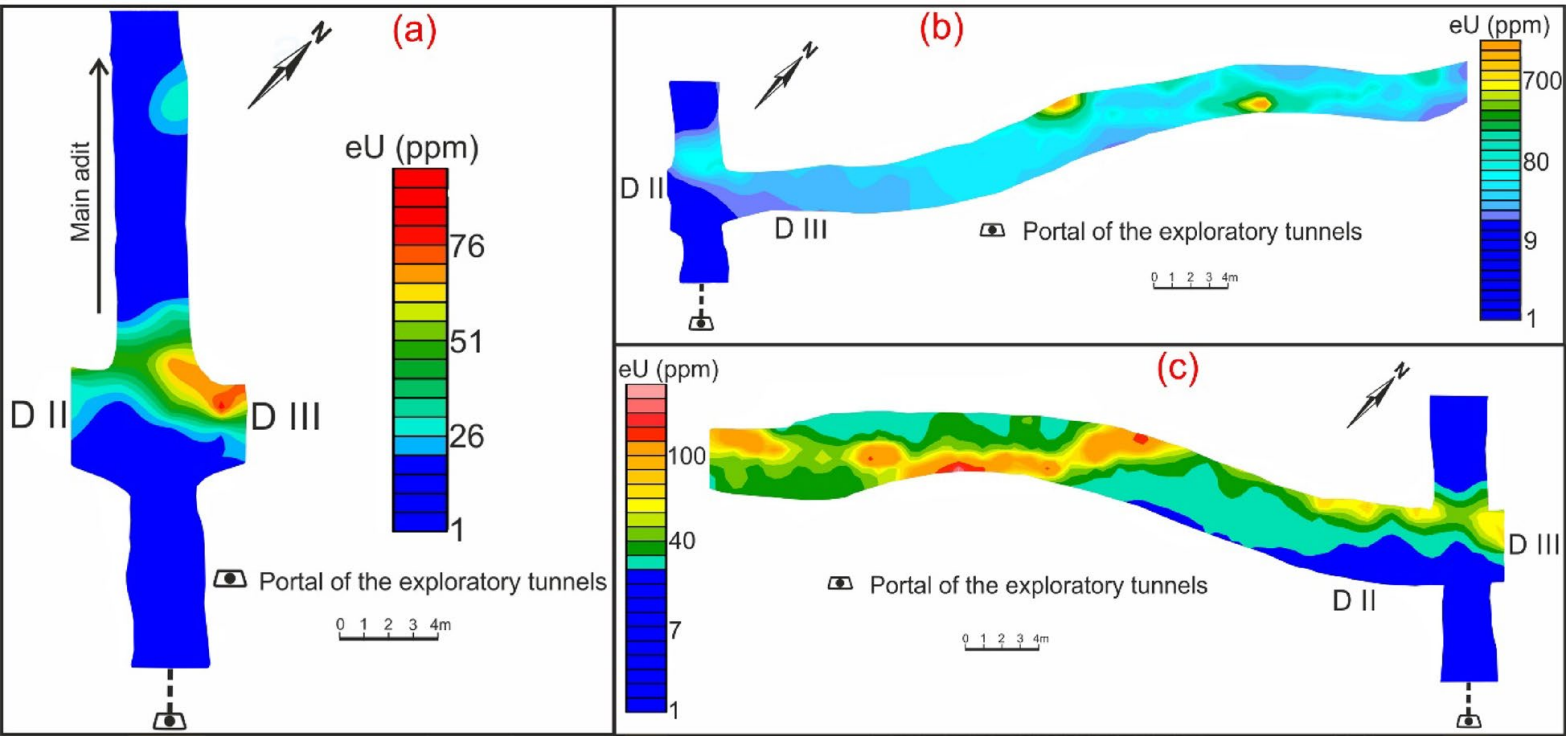
Contour maps of uranium concentration through main adit (**a**) and shear zone (drift# DIII (**b**) & DII (**c**)) of El-Erediya exploratory tunnels.

**Fig. 7:**
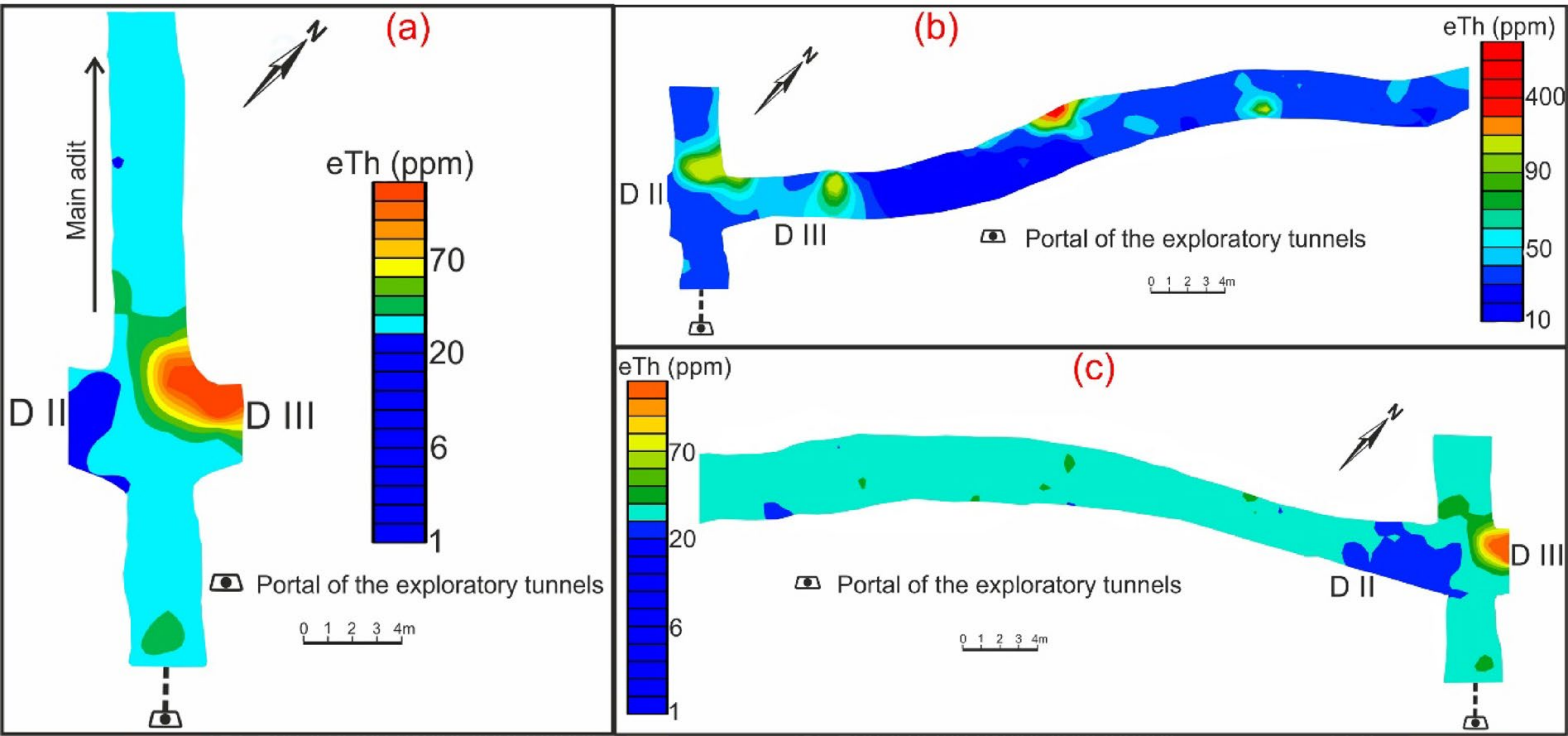
Contour maps of thorium concentration through main adit (**a**) and shear zone (drift# DIII (**b**) & DII (**c**)) of El-Erediya exploratory tunnels.

**Fig. 8:**
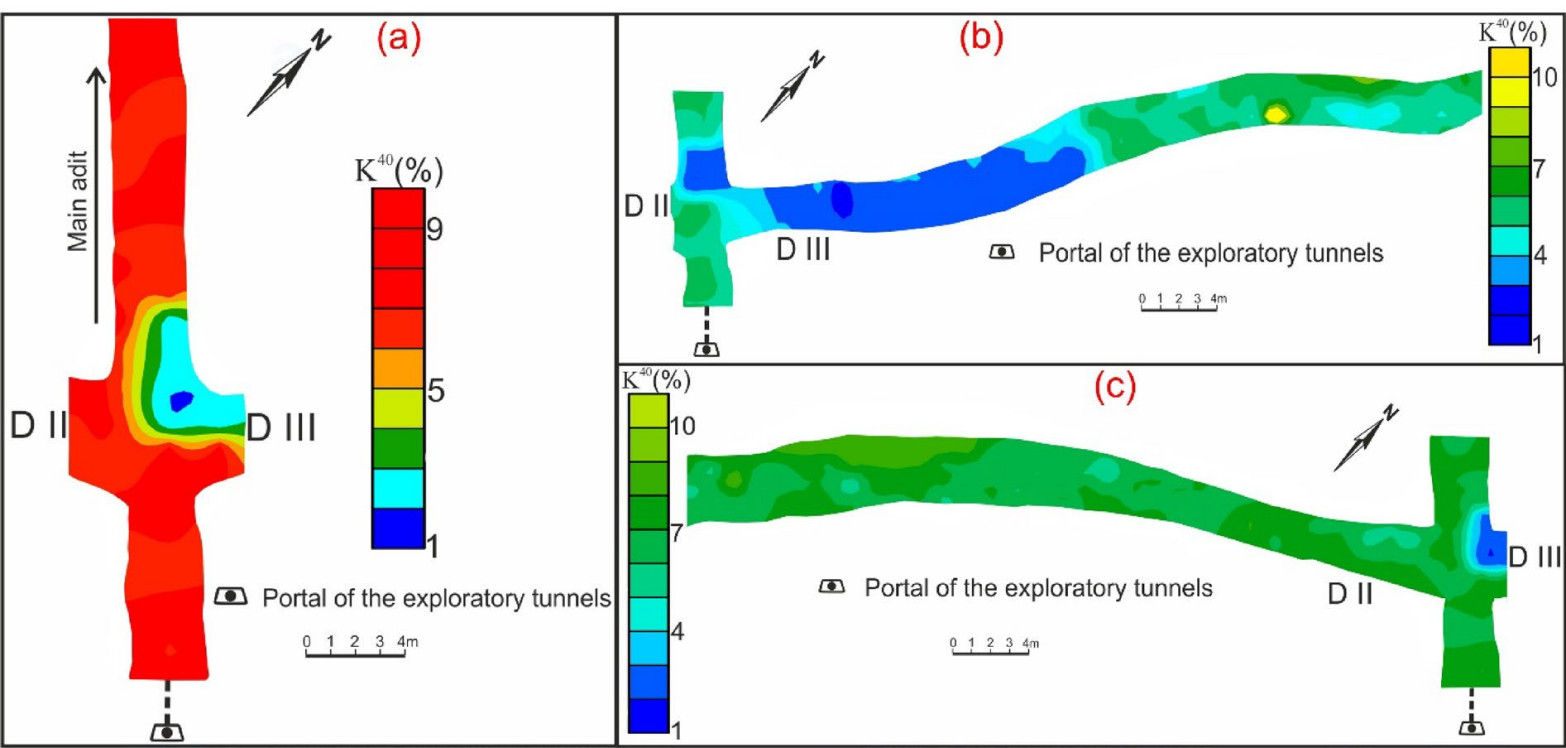
Contour maps of K^40^ concentration through main adit (**a**) and shear zone (drift# DIII (**b**) & DII (**c**)) of El-Erediya exploratory tunnels.

The measured radiometric data were mapped and presented in the Figs. 6, 7 and 8, while the statistical analyses values are summarized in the accompanying Tables 1 and 2.

**Table 1:** Distribution of eU, eTh, and K^40^ in pink granite through the main adit at Erediya exploratory tunnels.

Location	Statistical elements	eU (ppm)	eTh (ppm)	K^40^ (%)	eTh/eU	(eTh/K) * 10^−4^
Main adit	Maximum	39.1	42.7	9.8	4.8	6.37
	Minimum	7.4	26.1	6	1.01	3.68
	Average	25	37.5	8.4	2.42	5.05
	Correlation coefficient	eTh and eU	0.04		–	–
		eU and K^40^	0.15		–	–

**Table 2:** Distribution of eU, eTh, and K^40^ through shear zone at El-Erediya exploratory tunnels.

Location	Statistical elements	eU (ppm)	eTh (ppm)	K^40^ (%)	eTh/eU
Shear zone	Maximum	2990.5	685.8	8	7.4
	Minimum	12.9	16.3	0	0.09
	Average	83.7	42.6	5.5	0.1

The average concentration of radioelements in El-Erediya pink granite is 25 ppm eU, 37.5 ppm eTh, and 8.4% K^40^. Comparing these concentrations with the normal average in granites, which is typically around (2–8 ppm eU, 10–30 ppm eTh, and 3.6–4.5% K^40^)^53,54^, it becomes clear that El-Erediya granite has higher concentrations of radioelements than the normal average in granite (Figs. 6a, 7a, 8a). The El-Erediya pink granite has an eTh/eU ratio ranging from 1.01 to 4.8, with an average of 2.42. This average value of the current studied granite is below the normal crustal ratio (2.5–5)^55–57^ and it shows a weak correlation between eU and eTh (0.04), indicating that the granite may have undergone to some degree of U enrichment. In addition, the El-Erediya pink granite shows an average value of eTh/ K^40^ ratio of 5.05 × 10^−4^ and a week correlation between eTh and K^40^ (0.15). Comparing this value with normal value of unaltered rock (3 × 10^−4^ to 5 × 10^−4^)^58,59^, it can be suggested that El-Erediya granite corresponds to the normal crustal value of the unaltered lithology.

On the other hand, the shear zone is characterized by a higher eU and eTh concentration than that recorded in pink granite at the main adit. The radioelement concentration through this shear zone ranges from 12.9 to 2990.5 ppm eU (avg., 83.7 ppm eU), from 16.3 to 685.8 ppm eTh (avg., 42.6 ppm eTh), and from 0 to 8% K^40^ (avg., 5.5% K^40^) (Figs. 6b, c, 7b, c, 8b, c). It has a wide eTh/eU ratio range from 0.09 to 7.4 (avg., 0.1), indicating that the post magmatic processes (enrichment & depletion) played an important role in mobilization and redistribution of U^60^. The range of eTh/eU of the hydrothermal uranium mineralizations (hydrothermal uranium enrichment) is “2.5 > eTh/eU > 0.1”^61–63^, while value of eTh/eU ≤ 0.1 belongs to supergene uranium mineralizations (supergene uranium enrichment)^64,65^. According to the average and minimum value of eTh/eU ratio, the uranium enrichment processes occurred due to hydrothermal (non-magmatic source) and supergene processes.

### Distribution of radioelements within FCM clusters through main adit and shear zone

**Fig. 9:**
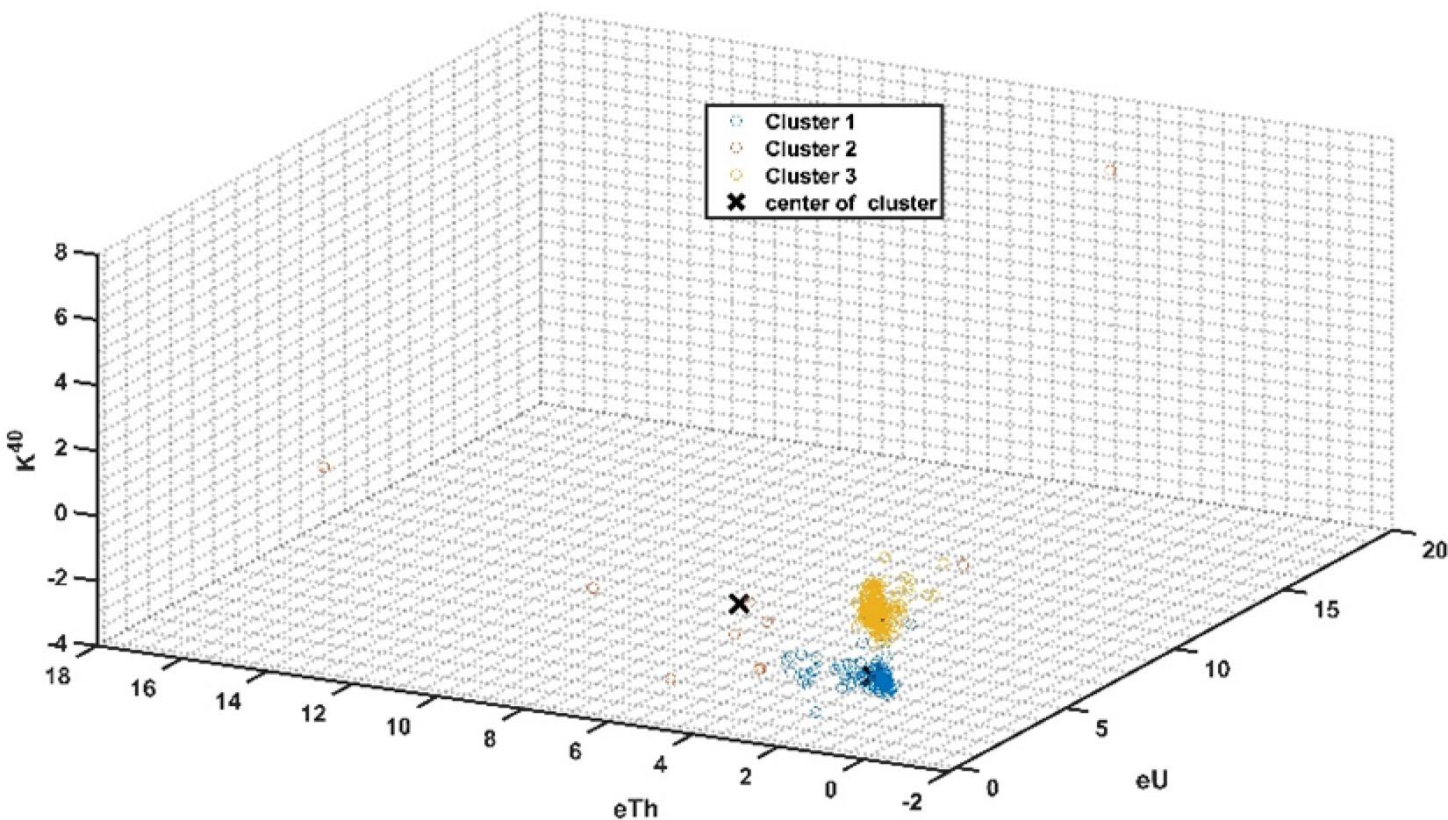
The clustering diagram of radioelements measurements obtained from FCM clustering analysis. Three clusters are shown.

**Fig. 10:**
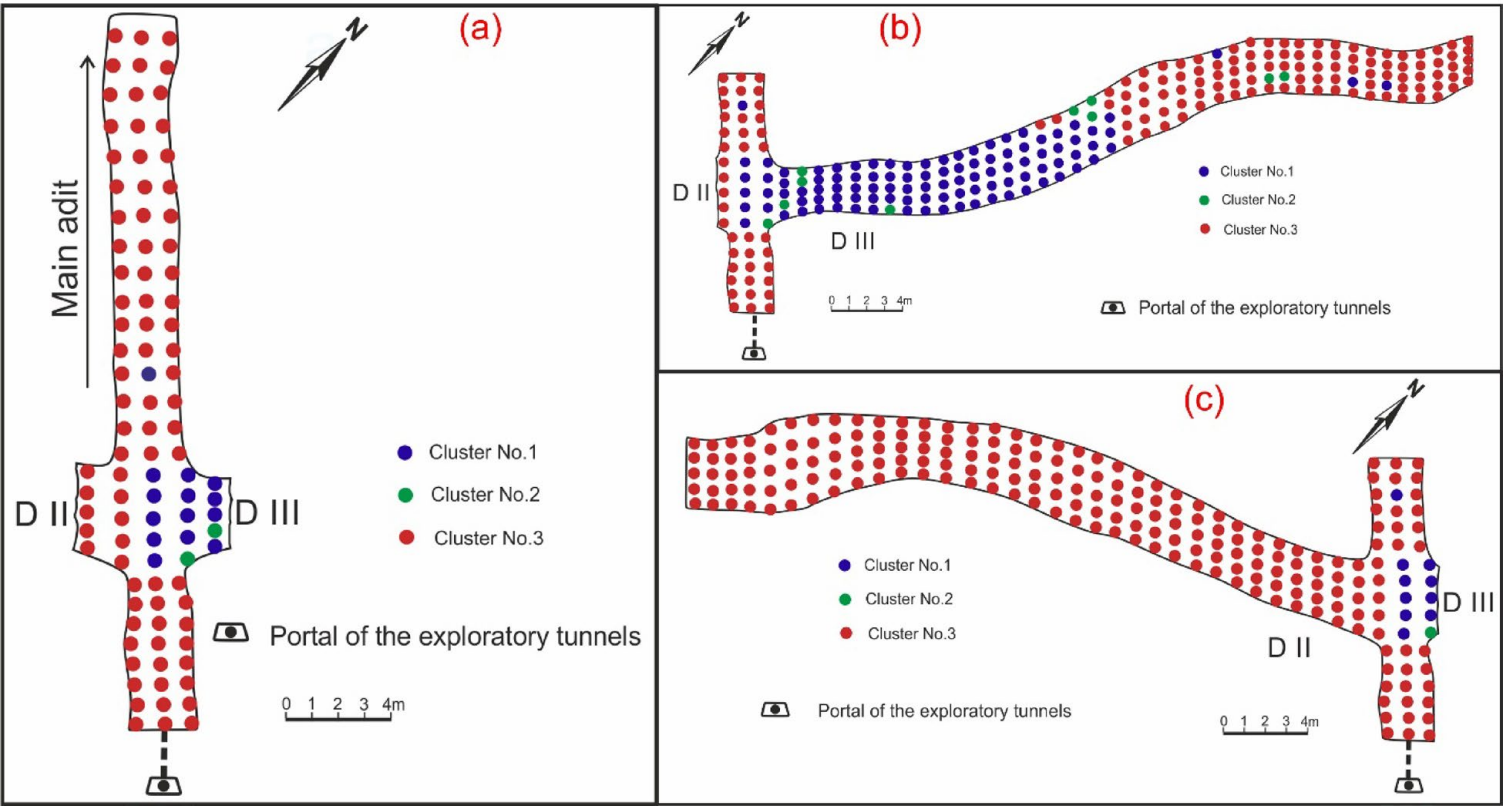
The cluster map showing the locations of clusters at the roof of the main adit (**a**), drift# DIII (**b**), and drift# DII (**c**) of El-Erediya exploratory tunnels.

**Fig. 11:**
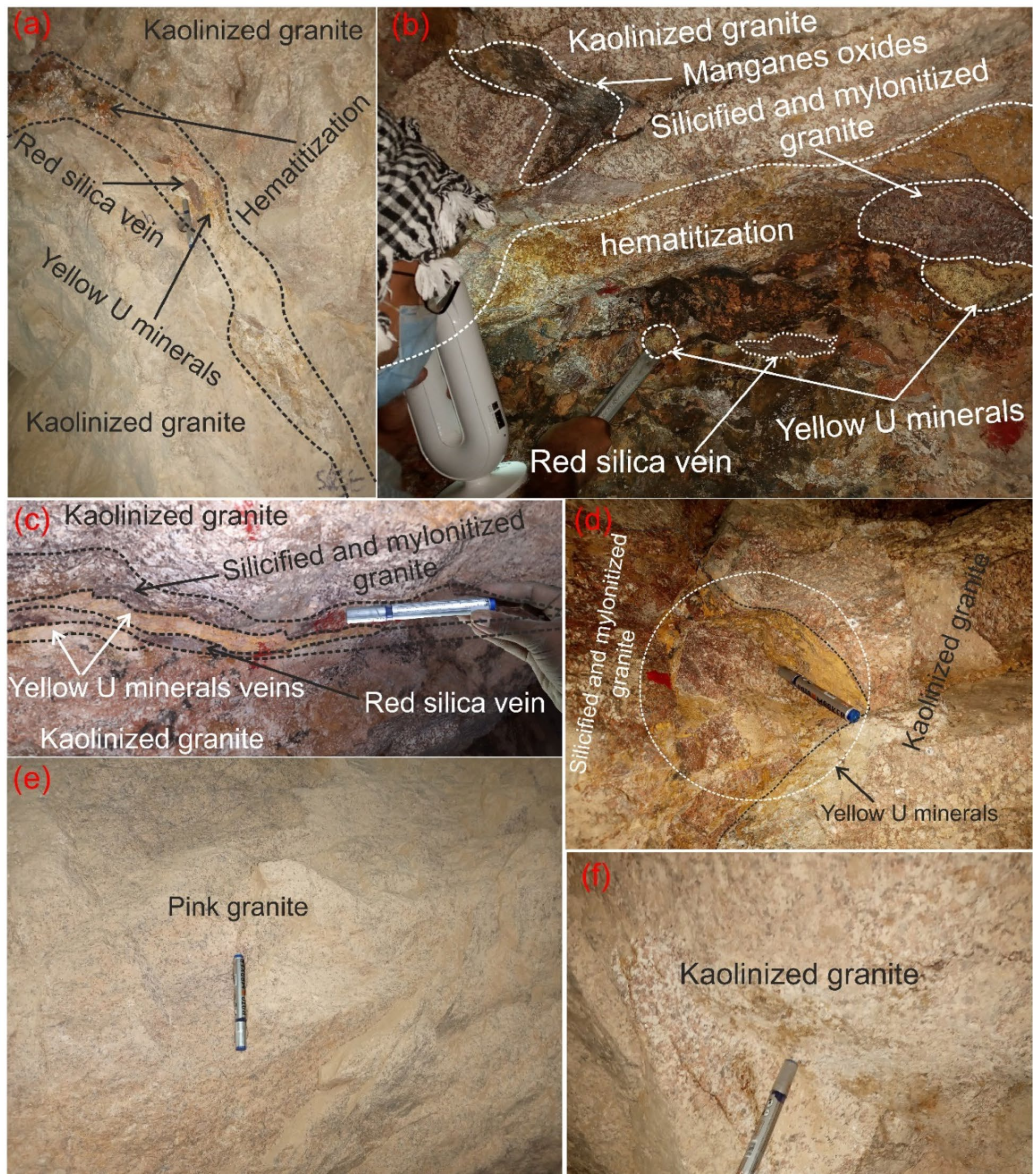
Field photographs show geologic features of different clusters (**a**–**c**) assigned to cluster No. 2; (**d**, **e**) assigned to cluster No. 3; (**f**) assigned to cluster No. 1 through of El-Erediya exploratory tunnels.

Three clusters were identified based on the fuzzy C-means clustering analysis (FCM) calculated for radioelements measurements along El-Erediya exploratory tunnels (Fig. 9). Out of these, three clusters (cluster No. 1, No. 2, and No. 3) were found in the main adit and drift No. III, while cluster No. 3 were located in drift No. II of El-Erediya shear zone (Fig. 10). Cluster No. 2 is distinguished by very high radioelements (U, and Th) concentrations, while cluster No. 1 is characterized by low radioelements concentrations except thorium values compared with cluster No. 3 (Table 3). Cluster No. 2 has a high concentration of radioelements ranging from 29.6 to 2990.5 ppm eU (avg., 629.8 ppm eU), from 59.3 to 685.8 ppm eTh (avg., 224.4 ppm eTh), and from 1.1 to 8% K^40^ (avg., 4.1% K^40^). This cluster is associated with red silica veins, silicification, hematitization, manganese oxides and mylonitization, (Fig. 11a–c) which provide a suitable environments and traps for capturing, protecting and accommodating U mineralizations^23,25,66–72^. Thorium is characterized by very low mobility in both the hypogene and supergene solutions^73^, which suggests that the high concentration of eTh in this cluster is due to successive pulses of magmatic mineralized solutions that are related to red silica veins^74^ and this enrichment of Th is represented by thorium-rich minerals (e.g., thorite, zircon, and xenotime)^75^ (see Fig. 11a–c). The high content of K^40^ in this cluster is correlated to leached K from El-Erediya hydrothermal altered felsic pink granite (kaolinized granite), concentrated and enriched along fractures that were invaded by silica veins^76–78^ (see Fig. 11b, c). Santaguida et al. found that K-feldspar alterations were followed by the presence of widespread sericitization and sulfide minerals^79^, which demonstrate that cluster No. 2 (rich in k-feldspar alteration) is also rich in sulfide minerals (e.g., pyrite, chalcopyrite, and galena). Cluster No. 3 has a high content of eU and K^40^ except eTh, ranging from 7.4 to 680.3 ppm eU (avg., 57.9 ppm eU), from 23.2 to 58.7 ppm eTh (avg., 35.5 ppm eTh), and from 4.6 to 9.8% K^40^ (avg., 7% K^40^). In this cluster, the high content of eU is accompanied by silicification and mylonitization that protected U mineralizations (Fig. 11d), while the high concentration of K^40^ is related to unaltered El-Erediya younger pink granite^53,54,78^ (Fig. 11e). The concentration of eU and K^40^ in cluster No. 1 is low compared with cluster No. 2, and No. 3, ranging from 15.5 to 113.4 ppm eU (avg., 52.4 ppm eU), and from 0 to 7.4% K^40^ (avg., 2.9% K^40^), while eTh concentration is high compared with cluster No. 3, ranging from 16.3 to 118.2 ppm (avg., 43.4 ppm). In this cluster, the low content of eU, and K^40^ is allied with kaolinized granite (kaolinization) due to the limited surface area of kaolinite reduces the number of available adsorption sites for U⁶⁺ ions present in the invading solutions^69^, while low K^40^ content due to released K from altered pink granite (kaolinized granite and /or argillic alteration)^80^ (Fig. 11f, and see Fig. 4). On the other hand, argillic facies are distinguished by the occurrence of some minerals such as pyrite, galena, gold, ilmenite, barite, celestine, magnetite, hematite, and goethite^81^.

**Table 3:** Distribution of eU, eTh, and K^40^ within clusters through main adit and shear zone at El-Erediya exploratory tunnels.

Location	Cluster number	Statistical elements	eU (ppm)	eTh (ppm)	K^40^ (%)
Main adit + shear zone	Cluster No. 1	Maximum	113.4	118.2	7.4
		Minimum	15.5	16.3	0
		Average	52.4	43.4	2.9
	Cluster No. 2	Maximum	2990.5	685.8	8
		Minimum	29.6	59.3	1.1
		Average	629.8	224.4	4.1
	Cluster No. 3	Maximum	680.3	58.7	9.8
		Minimum	7.4	23.2	4.6
		Average	57.9	35.5	7

### Petrographic characteristics of host rock within FCM clusters

**Fig. 12:**
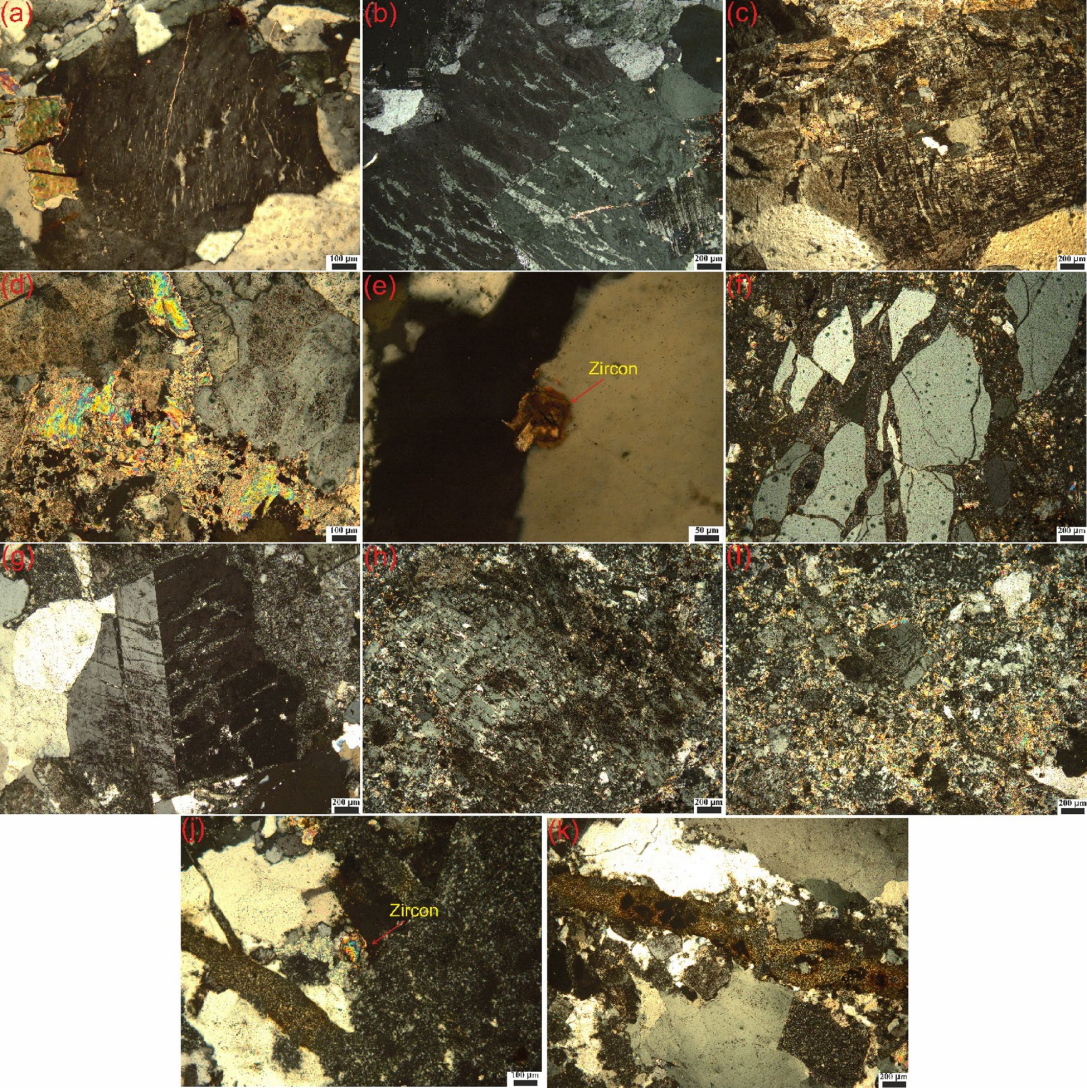
Photomicrographs of alkali-feldspar granite; [(**a**) wavy extinction of quartz, string perthite, and irregular flakes of biotite, (**b**) orthoclase perthite, and albite twining of plagioclase, (**c**) cross-hatched twining microcline, (**d**) primary flakes and secondary fan-shaped of muscovite, and (**e**) elbow twining of zircon] and their alterations; [(**f**) fragments and undulose extinction of quartz, (**g**) Partially silicification of orthoclase perthite, (**h**) partially kaolinization and silicification of perthite, (**i**) partially sericitization and silicification of perthite, (**j**) zircon crystal associated with silicification and hematitization, and (**k**) fractures filled with opaque minerals].

Besides ground radiometric measurements, Polarized Transmitted Light Microscope was applied to thin sections of representative samples from FCM clusters to identify silicate minerals and their alterations. Megascopically, the studied host granite rock (alkali-feldspar granite) is a medium-grained massive rock and pink in color; characterized by hypidiomorphic texture. Microscopically, alkali-feldspar granite is composed mainly of quartz (avg., 38.7%), potash feldspar (avg., 59.8% of the whole rock composition), with few crystals of plagioclase, biotite and muscovite. Quartz occurs as anhedral crystals, characterized by wavy extinction (Fig. 12a). Potash feldspar occurs as anhedral to subhedral of string perthite, orthoclase perthite, and microcline, which is characterized by cross-hatched twining (Fig. 12a–c). Plagioclase (An8) crystals are present and constitute approximately 1.1% of the rock composition; they are characterized by albite twining (Fig. 12b). Biotite occurs as irregular flakes characterized by weak pleochroism from pale brown to yellow color (Fig. 12a). Muscovite is forms primary flakes mottled by iron oxides or as fan-shaped secondary muscovite (Fig. 12d). Zircon occurs as euhedral prismatic crystals, exhibits elbow twining, coated by iron oxides and associating the quartz (Fig. 12e). Along the shear zone, alkali-feldspar granite is mylonitized and affected by silicification, kaolinization, hematitization, and sericitization processes. Quartz occurs as fractured crystals that fragmented and separated into angular grains (Fig. 12f). It is characterized by wavy and undulose extinction. The rock is enriched by the secondary silica that was produced by silicification of the feldspars. Most of the potash feldspars are partially and completely silicified and kaolinized or sericitized (Fig. 12g–i). Plagioclase is nearly absent due to the alkalinity of the rock and alteration of the plagioclase to clay minerals and silicification. Zircon occurs as euhedral crystals of prismatic form, characterized by second order interference color and zonation, associated with silicification (secondary silica), quartz and iron oxides (Fig. 12j). Opaque minerals present as mineralization filling the fractures or as hematite adsorbed by the amorphous silica (Fig. 12k).

### Identified opaque minerals of FCM clusters

**Fig. 13:**
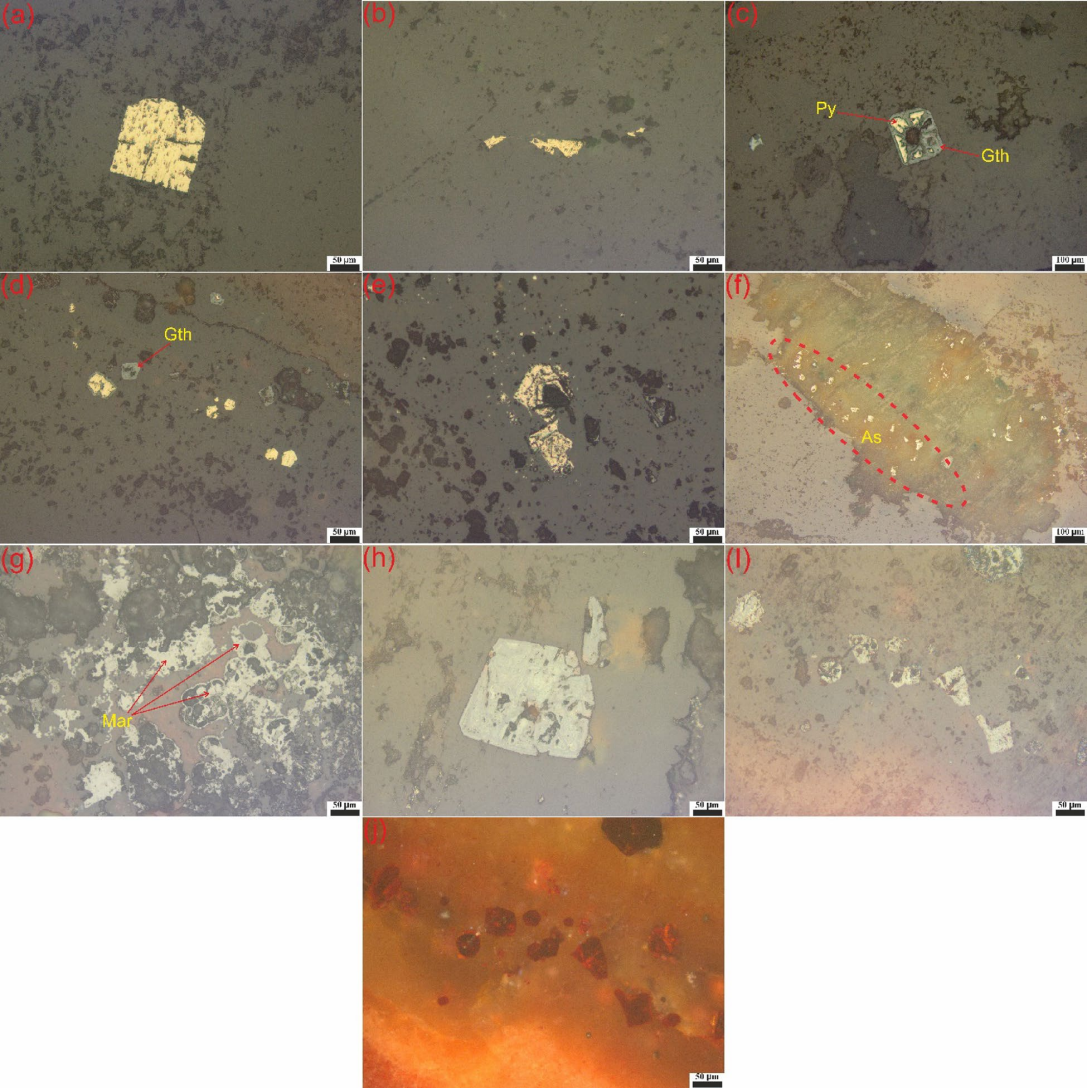
Photomicrographs of the different opaque minerals: (**a**) Pyrite (Py) occurred as domal form or (**b**) as cavity filling within silicified granite. (**c**) Partially altered Pyrite and (**d**) completely altered pyrite to goethite (Gth). (**e**) Chalcopyrite occurs as an anhedral crystal. (**f**) Arsenopyrite (As) disseminates in the prismatic crystal of feldspar. (**g**) Marcasite (Mar) appears as anhedral crystals with coin form. (**h**) Magnetite occurs as euhedral with bipyramidal form. (**i**, **j**) Magnetite crystals are replaced partially or completely with hematite (martitization).

Besides petrographic investigation, Polarized Reflected Light Microscope was conducted on polished sections of representative samples to characterize opaque minerals associated with radioactive minerals. Most of the opaque minerals that were identified by ore-microscopic investigation from clusters No. 1 and No. 2 include pyrite, chalcopyrite, arsenopyrite, marcasite, magnetite, goethite and hematite. Pyrite is the most abundant opaque mineral, occurs as well-formed crystals, exhibit the pyritohedron (domal form) or cavity filling within silicified granite (Fig. 13a, b). Some of pyrite crystals are altered partially or completely to goethite (Fig. 13c, d). The goethite (iron oxyhydroxide) in the shear zone acts as a reducing medium, converting mobile (U^+6^) to the insoluble (U^+4^)^83^. Chalcopyrite occurs as an anhedral crystal, disseminated in feldspar crystals (Fig. 13e). Arsenopyrite is found as subhedral to anhedral monoclinic crystal disseminated in the prismatic crystal of feldspar (Fig. 13f). Marcasite appears as anhedral orthorhombic crystals with coin form (Fig. 13g). Magnetite occurs as euhedral to subhedral crystals of cubic system with bipyramidal form (Fig. 13h). Most of magnetite crystals are replaced partially or completely with hematite (martitization), showing red ochre (Fig. 13i, j). Magnetite and its alteration product “hematite” played a vital role in the fixation of uranium from its uranyl-solutions during the invaded hydrothermal activities^84,85^. Uranyl-minerals and sulfide minerals are precipitated together from the hydrothermal solution due to the availability of reducing environment that comes from the presence of (H_2_S) and the increasing of S^+2^ that act as reducing agent for uranyl-ions^85,86^ (e.g., betafite).

### Separated radioactive minerals from FCM clusters

**Fig. 14:**
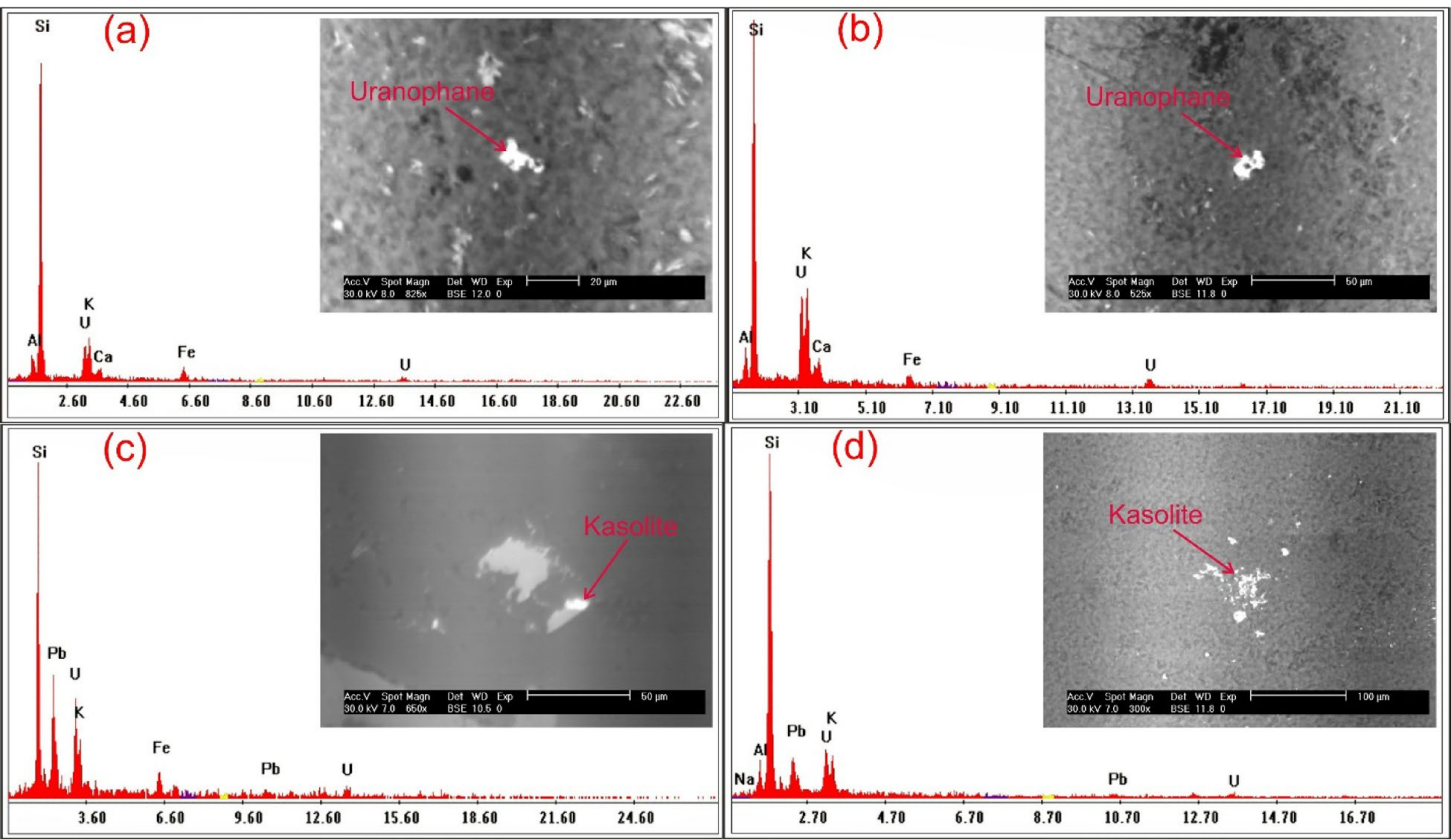
ESEM images of uranophane (**a**, **c** assigned to cluster No. 2) & kasolite (**b**, **d** assigned to cluster No. 3).

**Fig. 15:**
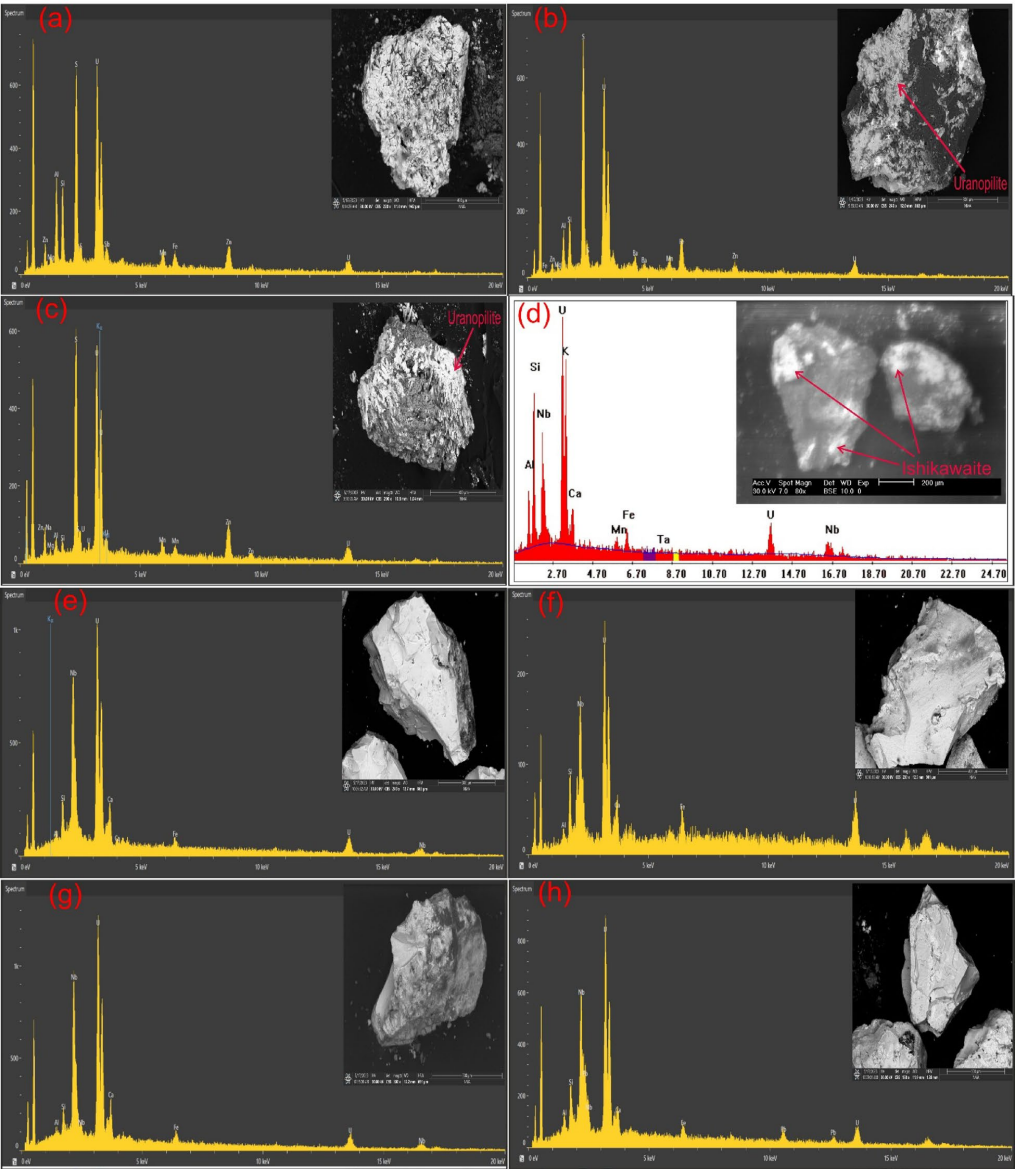
ESEM images of uranopilite (**a**–**c** assigned to cluster No. 1, No. 2, and No. 3, respectively), ishikawite (**d** assigned to cluster No. 2), betafite (**e**–**g** assigned to cluster No. 1, No. 2, and No. 3, respectively), and plumbobetafite (**h** assigned to cluster No. 2).

**Fig. 16:**
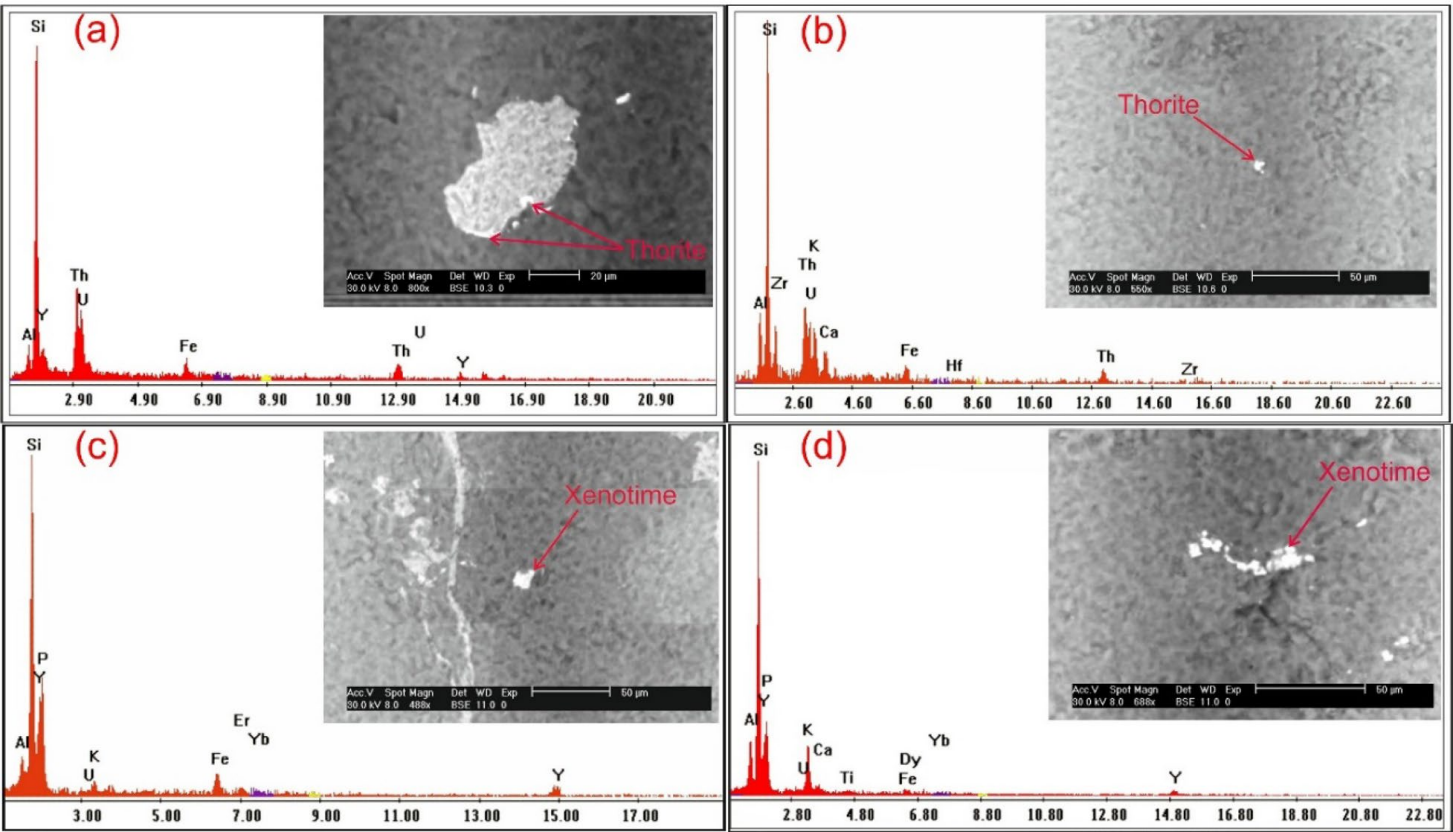
ESEM images of thorite (**a**, **b** assigned to cluster No. 1, and 2, respectively) & xenotime (**c**, **d** assigned to cluster No. 2, and 3, respectively).

**Fig. 17:**
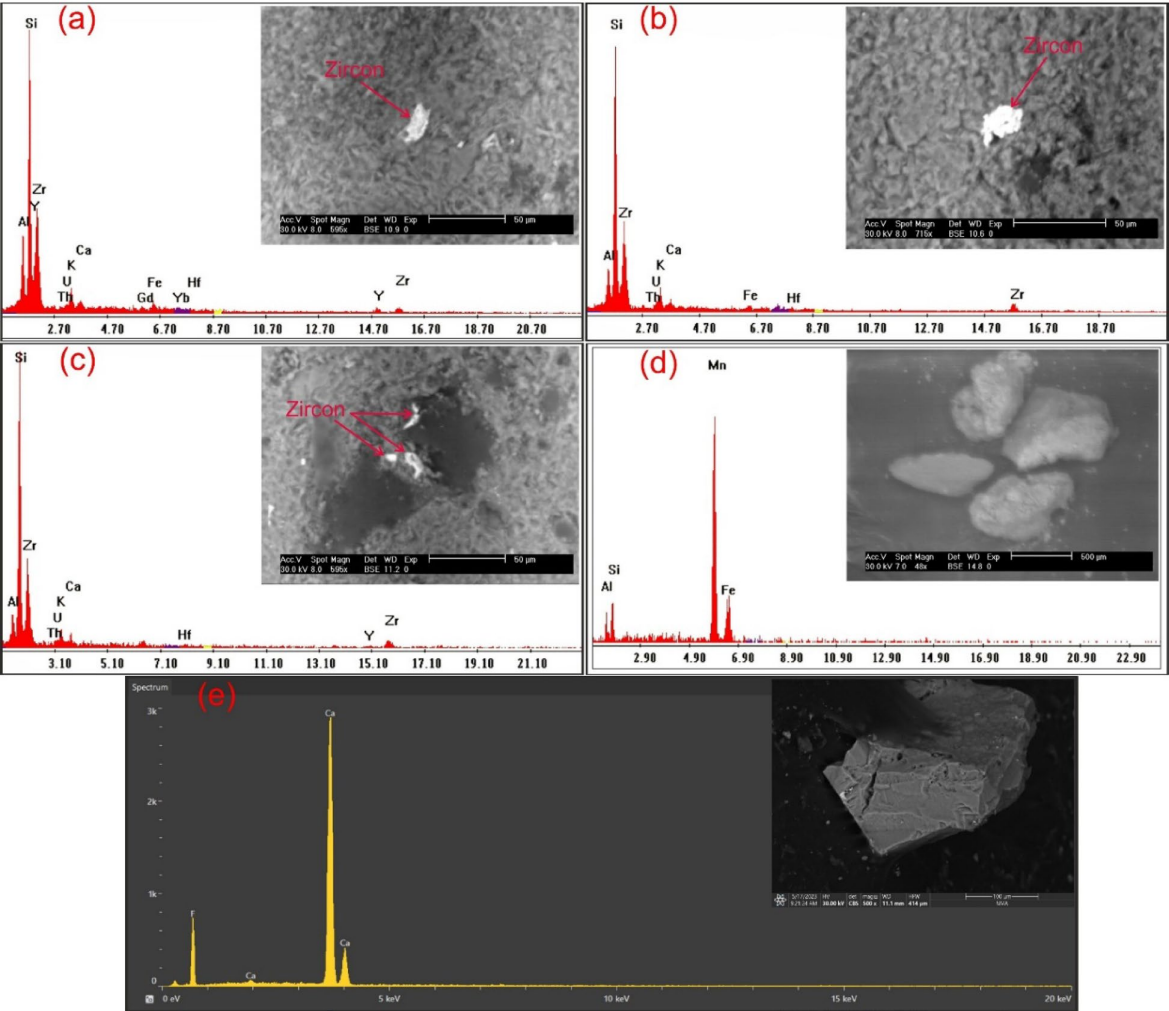
ESEM images of zircon (**a**–**c** assigned to clusters No. 1, 2, and 3, respectively), pyrolusite (**d** assigned to cluster No. 3), and fluorite (**e** assigned to cluster No. 3).

**Fig. 18:**
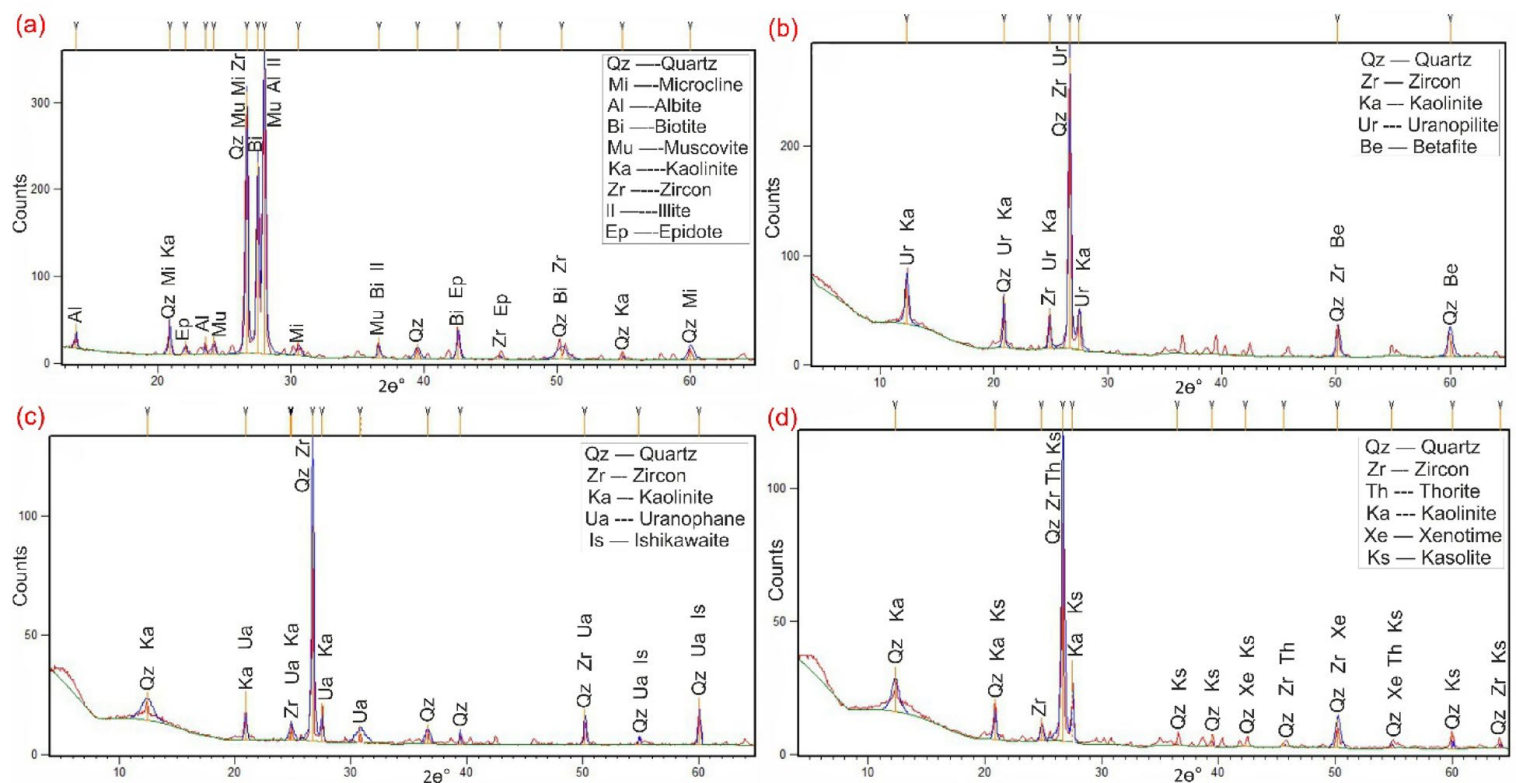
X-Ray Diffraction (XRD) pattern of El-Erediya alkali-feldspar granite (**a**) and their alterations (**b**–**d**) showing the bulk of mineralogy.

In addition to ground radiometric measurements, and petrographic, and ore-microscopic investigations, Environmental Scanning-Electron Microscope Energy Dispersive X-Ray analysis (SEM–EDX) were proceeded on separated mineral grains and thin sections of representative samples to define and determine the radioactive mineral assemblages. This analysis revealed the presence of radioactive minerals of magmatic origin, including thorite, and of hydrothermal origin, such as uranopilite, betafite, plumbobetafite, ishikawaite, xenotime, and zircon. In addition, supergene uranium minerals were detected, primarily uranophane and kasolite (Table 4). Uranophane and kasolite were found in cluster No. 2 and 3, indicating that these two clusters underwent uranium enrichment through supergene process^87,88^. According to EDX data, uranophane is composed of Si (up of 53.92 wt%), U (up to 39.81 wt%) and Ca (up to 3.29 wt%) with some Fe (up to 4.32 wt%) that may be related to hematitization processes (Fig. 14a, b), while the composition of kasolite is U (up to 41.05 wt%), Si (up to 52.88 wt%) and Pb (up to 15 wt%) (Table. 4, Fig. 14c, d). Uranopilite, ishikawite, betafite, and plumbobetafite were separated from clusters No. 1, 2, and 3, which indicates that these clusters suffered uranium enrichment by hydrothermal processes. Occasionally, uranopilite is associated with sulfide minerals, as the EDX analysis shows that uranopilite consists mainly of U (up to 56.1 wt%) and S (up to 19.8 wt%). Some types are enriched by iron oxides up to 6.1 wt% and could be categorized as Fe-uranopilite, while others are rich in sodium (4.1 wt%) and categorized as sodic uranopilite (Na-uranopilite) (Table 4, Fig. 15a–c). The EDX analysis shows that ishikawite consists of 50.58 wt% U, 2.90 wt% Ca, 2.54 wt% Fe and 22.56 wt% Nb equivalent to 225,600 ppm Nb (Table 4, Fig. 15d). Nb is an incompatible trace element that is mostly concentrated in residual melt^89,90^. The concentrations of Nb in the continental crust are 25 ppm^92^, but this mineral has a very high content of Nb, indicating that this mineral was subjected to Nb enrichment by several pluses of magmatic fluids. On the other hand, betafite contains U (up to71.7 wt%), Ca (up to 5.8 wt%), Nb (up to 19.7 wt%), Si (up to 5.3 wt%) and Fe (up to 3.8 wt%) as shown in EDX analysis (Table 4, Fig. 15e–g). This mineral is unstable and altered to kasolite, indicating that the prevailing environment is alkaline environment^93^. Moreover, the EDX data of plumbobetafite clarified presence of Pb (12 wt%) besides the other constituents of betafite as U (58.9 wt%), Nb (16 wt%), Ca (3.1 wt%), Si (5 wt%) and Fe (2.8 wt%) (Table 4, Fig. 15h). Thorite was separated from cluster No. 1, and 2, while xenotime was separated from cluster No. 2, and 3. The presence of thorite mineral (Th-rich mineral) indicate magmatic origin due to Th being a strong incompatible trace element because its high charge/radius ratio, which is mostly concentrated in residual magmatic solutions^89–92^, commonly shares a uniform crystallized site with REE in some REE minerals (e.g., monazite)^94^ and its liberation from its host rocks is very poor by hydrothermal solution. According to EDX analysis, the separated thorite mineral consists mainly of Th (up to 42.29 wt%) (Table 4, Fig. 16a, b). When comparing this concentration with the concentration of Th of thorite that separated from granitic rocks, which is typically around (25–80 wt%)^95–98^, it becomes clear that the origin of the separated thorite mineral is magmatic. Additionally, the presence of other trace elements (U = 8.55 wt% and Zr = 1.29 wt%) alongside Hf = 1.69 wt%, as well as the difference in Th concentration between the two thorite minerals, may be attributed to various metasomatism processes. The EDX data shows that xenotime contains Y (up to 26.31 wt%), P (up to 14.01 wt%) and U (up to 3.39 wt%) with some REEs (Er = 4.93 wt%, Yb = 5.15 wt%, Y = 26.31 wt% and Dy = 2.16 wt%) (Table 4, Fig. 16c, d). Zircon was detached from clusters No. 1, 2, and 3, while fluorite and pyrolusite were detached from cluster No. 2. The EDX data reflect the chemical composition of zircon of as Zr (up to 33.59 wt%), Si (up to 45.98 wt%) and Hf (up to 3.01 wt%) with some REEs (Y = 14.24 wt%, Gd = 1.61 wt%, and Yb = 3.22 wt%). Some crystals are metamectized due to the presence of radioelements (U = 4.19 wt% and Th = 1.50 wt%) (Table 4, Fig. 17a–c). The Th/U ratio of hydrothermal origin zircon is “0.5 > Th/U ≤ 0.1”^99–102^. The average value of Th/U of zircon minerals is 0.36, indicating hydrothermal origin of zircon. The EDX analysis shows that pyrolusite consists of 69.81 wt% Mn, and 5.93 wt% Fe, while fluorite consists of 44 wt% Ca, and 56 wt% F (Fig. 17d, e). Fluorite is considered to be a fixating agent of uranium from fluorite-uranium complex phases^85,103,104^. Moreover, the occurrence of manganese mineral (pyrolusite) plays an important role in immobilizing and capturing uranium from invading hydrothermal solutions^105,106^. The sulphate contents of uranopilite are thought to be derived from the supergene oxidation of sulphide minerals (e.g., oxidation of pyrite into goethite), and played an important role in the formation of secondary U-mineralization. Also, Energy-Dispersive X-Ray (EDX) analyses of these minerals were corroborated by X-Ray Diffraction (XRD) analyses conducted on the host rocks of various clusters (Fig. 18a–d).

**Table 4:**
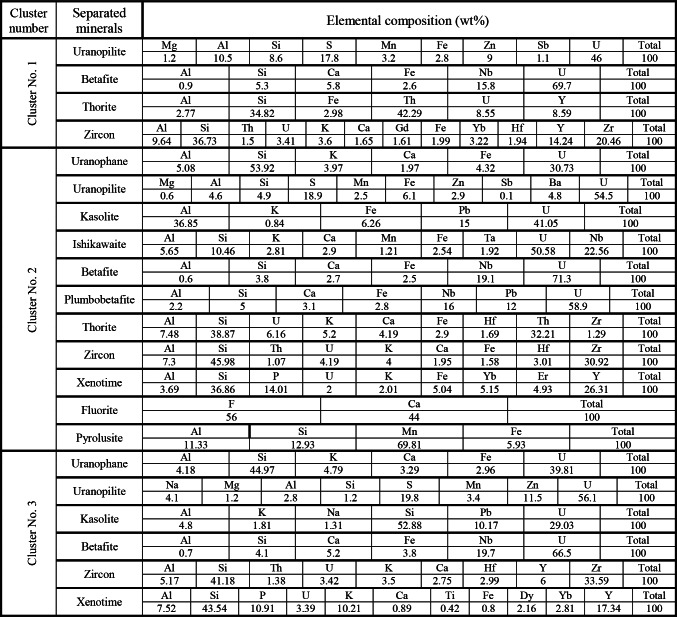
EDX analysis of the separated radioactive minerals from clusters within main adit and shear zone at El-Erediya exploratory tunnels.

## Conclusion

This study provides a detailed radiometric and mineralogical characterization of the main adit and the shear zone within the El-Erediya exploratory tunnels, one of the most promising radioactive mineralization in the Eastern Desert of Egypt, revealing significant variations in radioelement concentrations (eU, eTh, and K^40^) linked to distinct alteration zones and mineral assemblages. The implementation of fuzzy C-means clustering analysis (FCM) on ground gamma ray spectrometric measurements, enabled us to identify three distinct radiometric clusters associated with various hydrothermal alterations and mineralization styles. Furthermore, mineralogical investigations confirmed that the identified minerals have both magmatic and post-magmatic (hydrothermal and supergene) origins.

The integration of radiometric and mineralogical study offers valuable insights into the spatial distribution of radioactive minerals and their geological controls, establishing a foundation for advanced exploration to understand and exploit the El-Erediya shear zone mineralization.

## Data Availability

The data supporting this study’s findings are available from the corresponding author upon reasonable request.
